# DPPIV promotes endometrial carcinoma cell proliferation, invasion and tumorigenesis

**DOI:** 10.18632/oncotarget.14412

**Published:** 2017-01-02

**Authors:** Xiaoqing Yang, Xinhua Zhang, Rongrong Wu, Qicheng Huang, Yao Jiang, Jianbing Qin, Feng Yao, Guohua Jin, Yuquan Zhang

**Affiliations:** ^1^ Department of Obstetrics and Gynecology, Affiliated Hospital of Nantong University, NanTong, Jiangsu 226006, People's Republic of China; ^2^ Department of Anatomy, Nantong University, Nantong, JiangSu 226000, People's Republic of China

**Keywords:** endometrial carcinoma, dipeptidyl peptidase IV, sitagliptin, hypoxia-inducible factor 1a, vascular endothelial growth factor A

## Abstract

Dipeptidyl peptidase IV (DPPIV), also known as CD26, is a 110-kDa cell surface glycoprotein expressed in various tissues. DPPIV reportedly plays a direct role in the progression of several human malignancies. DPPIV specific inhibitors are employed as antidiabetics and could potentially be repurposed to enhance anti-tumor immunotherapies. In the present study, we investigated the correlation between DPPIV expression and tumor progression in endometrial carcinoma (EC). DPPIV overexpression altered cell morphology and stimulated cell proliferation, invasion and tumorigenesis *in vitro* and *in vivo*. These effects were abrogated by DPPIV knockdown or pharmacological inhibition using sitagliptin. DPPIV overexpression increased hypoxia-inducible factor 1a (HIF-1a) and vascular endothelial growth factor A (VEGFA) expression to promote HIF-1a-VEGFA signaling. Our results indicated that DPPIV accelerated endometrial carcinoma progression and that sitagliptin may be an effective anti-EC therapeutic.

## INTRODUCTION

Endometrial carcinoma (EC) is the most common gynecologic malignancy in women in western countries and the fourth most common cancer among women in the U.S. Both EC incidence and patient mortality rates are increasing, with 54,870 new cases and 10,170 deaths reported in 2015 [1]. Although Asian women have a comparatively lower EC risk, incidences of EC in China and Japan are also on the rise [2, 3]. The 5-year patient survival rate for well-differentiated EC is 96% in the early stage, but only 67% when accompanied by local or distant metastasis and 17% in poorly differentiated cases [4]. Early diagnosis and identification of new therapeutic targets may improve EC patient prognoses.

Rising rates of obesity and diabetes, two well-known risk factors in EC development and predictors of poor patient outcome, may be contributing to rising EC incidences [5, 6]. Glucose is a major energy source for cell growth and invasion, and treatments targeting glucose metabolism may be efficacious against EC [7–9]. Dipeptidyl-peptidase IV (DPPIV) inhibitors have been approved for treating patients with type 2 diabetes mellitus. These so-called gliptins increase incretin levels and thereby prolong post-prandial insulin action [10], potentially linking obesity to metabolic syndromes [11, 12].

DPPIV, also known as CD26, is a ubiquitously expressed 110-kDa glycoprotein [13] that binds numerous peptides, including adenosine deaminase and extracellular matrix proteins [14–16]. As a serine protease, DPPIV cleaves numerous substrates and is involved in intracellular signaling and immune cell activation. DPPIV expression is a cancer stem cell (CSC) marker in human malignancies such as colorectal cancer, chronic myeloid leukemia and malignant mesothelioma [17–19]. Some studies report that DPPIV acts as a tumor suppressor in melanoma, ovarian carcinoma, prostate cancer and cervical carcinoma cells [20–23]. In endometrial adenocarcinoma, DPPIV was expressed in normal endometrial glandular cells and EC [24, 25], although its contributions to tumorigenesis were not studied.

The present study investigated the relationship between DPPIV expression and malignancy in EC cells. Our results demonstrated that DPPIV overexpression induced cell morphological changes and stimulated cell proliferation, invasion and tumorigenesis *in vitro* and *in vivo*. These effects were abrogated by DPPIV knockdown and pharmacological inhibition by sitagliptin. DPPIV overexpression increased hypoxia-inducible factor-1α (HIF-1α) and vascular endothelial growth factor A (VEGFA) expression to promote HIF-1α-VEGFA signaling. Our results indicate that DPPIV accelerated endometrial carcinoma progression and that sitagliptin may be an effective EC treatment.

## RESULTS

### Cell morphology and estrogen receptor 1 (ER1) and progesterone receptor 1 (PR1) expression were different in the originial EC cell lines

HEC-1A, HEC-1B and KLE cells exhibited an epithelial morphology, unlike Ishikawa and AN3CA cells, which express lower endogenous DPPIV levels and had a spindle/bipolar shape typical of fibroblasts (Supplementary Figure 1A–1E). With the exception of Ishikawa cells, estrogen receptor 1(ER1) and progesterone receptor 1 (PR1) mRNA was almost undetectable by qRT-PCR in mouse tumors derived from these cell lines (Supplementary Figure 1F). These data are in agreement with the ATCC and other cell banks.

### DPPIV expression in EC cell lines

DPPIV expression was evaluated in various EC cell lines by FACS, qRT-PCR and western blot (WB) analysis (Supplementary Figure 2). About 80% of HEC-1A and HEC-1B cells, but<2% of Ishikawa and AN3CA cells, expressed DPPIV. DPPIV expression was 65-, 69-, and 11-fold higher in HEC1A, HEC-1B and KLE cells, respectively, than in Ishikawa cells.

### DPPIV up- and downregulation induced morphological changes in Ishikawa, HEC-1B and AN3CA cells

**Figure 1:**
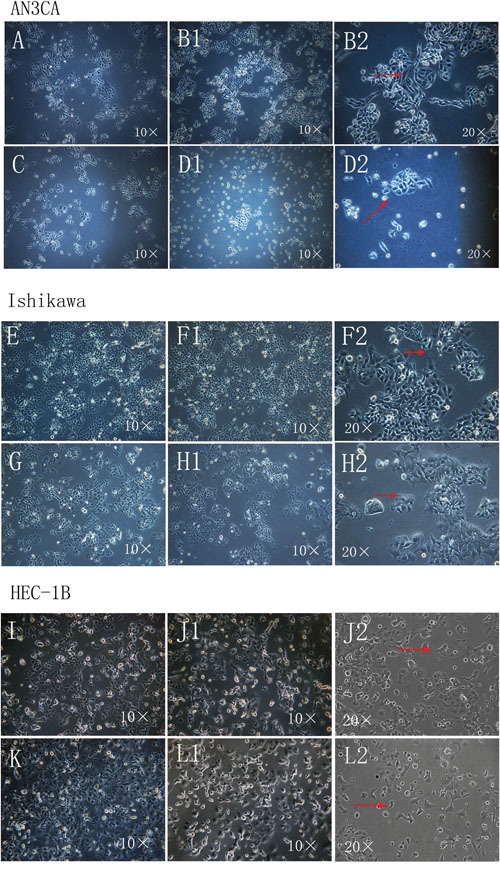
Morphological changes in Ishikawa, HEC-1B and AN3CA cells following DPPIV overexpression or knockdown **A–L.** Cells overexpressing DPPIV were actively growing and had more pseudopodia-like connections between cells (arrow) after transfection for 96 h and first passage 24 h later (B1 and B2, AN3CA cells; F1 and F2, Ishikawa cells; J1 and J2, HEC-1B cells). ShRNA-mediated DDPIV knockdown altered cell morphology; filamentous connections between cells were lost and cells became apoptotic (arrow) (D1 and D2, AN3CA cells; H1 and H2, Ishikawa cells; L1 and L2, HEC-1B cells); A, C, E, G, I and K negative controls). (Cell morphology was recorded using an IX71 microscopy system coupled to a DP73 digital camera).

We investigated the effect of DPPIV on EC cell morphology using Ishikawa(an ER- and PR-positive, well-differentiated cell line with low endogenous DPPIV expression cell), HEC-1B (an ER- and PR-negative, moderate-differentiated cell line with high endogenous DPPIV expression) and AN3CA cells (an ER- and PR-negative, undifferentiated malignant cell line with low endogenous DPPIV expression). Cells overexpressing DPPIV showed active growth and more pseudopodia-like connections between cells, whereas those in which DPPIV was knocked down by shRNA showed a loss of normal morphology, including filamentous intercellular connections. Knockdown cells grew in isolation, assuming a rounded, apoptotic shape relative to negative controls. These changes were particularly evident in AN3CA cells (Figure 1, Supplementary Figure 2B)

### DPPIV promotes EC cancer growth

**Figure 2:**
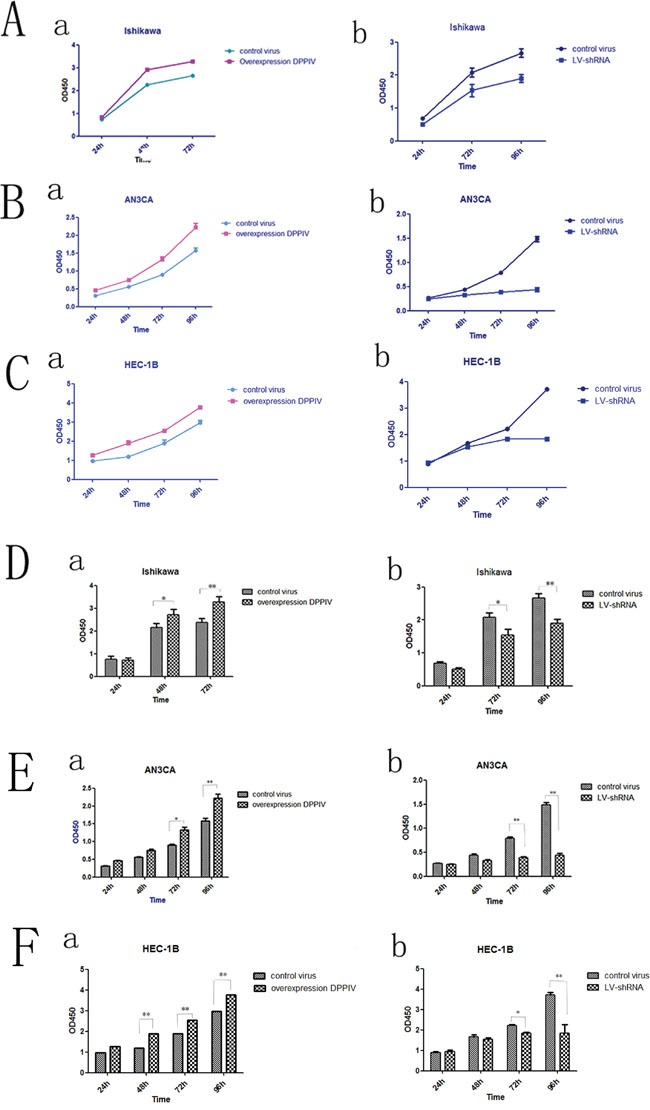
DPPIV overexpression increased cell proliferation in Ishikawa, HEC-1B and AN3CA cells, while shRNA-mediated DPPIV knockdown had the opposite effect **A-C.** DPPIV overexpression stimulates proliferation while DPPIV knockdown suppressed growth in Ishikawa (A), AN3CA (B) and HEC-1B (C) cells. **D-F.** Quantitative analysis of results shown in A-C results represent mean ± SD (n = 4). *P < 0.05; **P < 0.01 (Student's t test).

**Figure 5:**
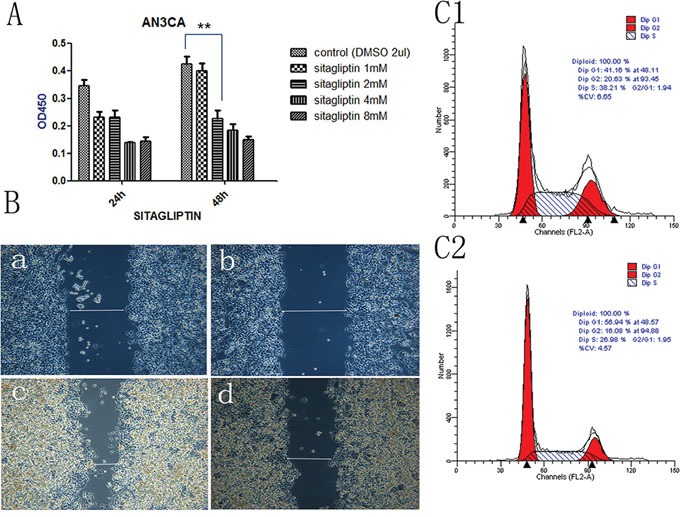
DPPIV inhibitor- sitagliptin suppresses cell proliferation, cell migration and cell cycle **A.** Cell viability is diminished by DDPIV inhibition. AN3CA cells were treated with the different concentrations DPPIV inhibitor-sitagliptin or left untreated and viability was evaluated 48 h later. Results are mean values ± SD (n = 4). **P < 0.001 (Student's t test). **B.** Cell migration in the absence (control) or presence of sitagliptin (1 mM). The graph is representative of three independent experiments. **C.** Cycle arrest at G0/G1 in the presence of sitagliptin (1 mM) (C2) or absence sitagliptin (C1).

We tested the effect of DPPIV on the growth of three EC cell lines, Ishikawa, HEC-1B and AN3CA cells. DPPIV overexpression increased cell proliferation 48 and 72 h after transfection in all three cell lines as compared to the negative control group (P<0.05; Figure 2A–2C), while shRNA-mediated DPPIV knockdown and pharmacological inhibition of DPPIV had the opposite effect. This was particularly evident in AN3CA cells (P<0.0001). When AN3CA cells treated with sitagliptin showed a dose-dependent decrease in cell growth (P<0.05). The half-maximal inhibitor concentration (IC50) of sitagliptin at 48 h was 1 mM (Figure 5A).

### DPPIV overexpression and knockdown alter cell adhesion and migration

**Figure 3:**
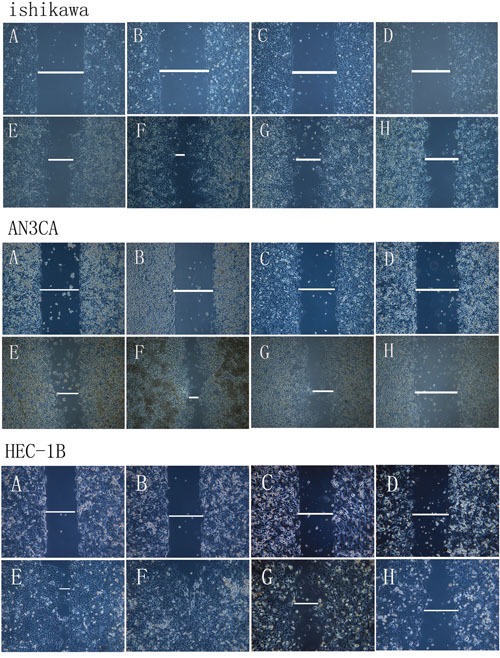
Analysis of migration in DPPIV-overexpressing or knockdown cells **A–H.** Ishikawa, HEC-1B and AN3CA cells were analyzed with the wound-healing assay at 0 h (A–D) and 24–48 h (E–H) after insert removal. A and E, overexpression control; B and F, DDPIV overexpression; C and G, shRNA control; D and H, LV-shRNA. Th migratory capacity of EC cells was enhanced by DPPIV overexpression (indicated by a line in F) and reduced by DPPIV knockdown (indicated by a line in H) especially in AN3CA cells after 24–48 h of culture.

We used an *in vitro* wound-healing model to assess the effect of DPPIV overexpression or inhibition on cell migration in Ishikawa, HEC-1B and AN3CA cells. DPPIV overexpression stimulated cell migration relative to controls, and this was abrogated by DPPIV knockdown, especially in AN3CA cells after 48 h (P<0.05; Figure 3). AN3CA cell migration was also inhibited by 48 h sitagliptin treatment (P<0.05; Figure 5B).

### DPPIV inhibition induces cell cycle arrest in EC cells

**Figure 4:**
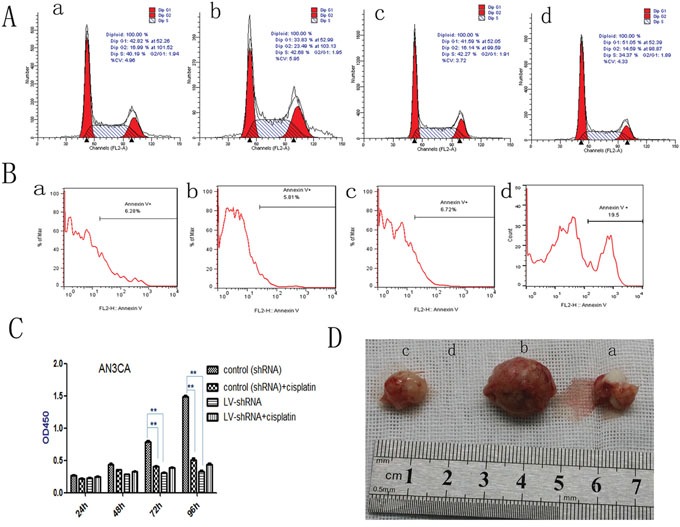
DPPIV inhibitor induces cell cycle arrest, induces apoptosis; DPPIV knockdown and the chemotherapy sensitivity test; DPPIV overexpression increases tumorigenicity **A.** DPPIV depletion induces cell cycle arrest at G0/G1, while DPPIV overexpression increases cells enter into S and G2 phase. **B.** DPPIV depletion induces apoptosis (B) in AN3CA cells, as determined by flow cytometry. **C.** DPPIV knockdown inhibited cell proliferation, similar to the effects of cisplatin. **P < 0.001, there were no synergistic effects associated with DPPIV knockdown and concurrent cisplatin treatment after 48, 72 and 96h. (P >0.05). The 95% confidence interval is (-0.887, -0.00129), (-0.960, -0.684), (-0.1560, -0.07248) respectively. **D.** DPPIV overexpression and knockdown compare with they control group at 8 weeks after injection. a, overexpression control; b, DDPIV overexpression; c, shRNA control; d, LV-shRNA. The graph is representative of three experiments.

We examined the role of DPPIV in the cell cycle in Ishikawa and AN3CA cells. DPPIV knockdown increased the G1 population from 41.59% to 51.05% and reduced the S-phase fraction from 42.27% to 34.37% in AN3CA cells. Conversely, DPPIV overexpression increased the percentage of cells in S and G2 phases (P<0.05; Figure 4A). Sitagliptin treatment induced cell cycle arrest 48 h after treatment in AN3CA cells, with an increase in the G1 population from 41.16% to 56.94% (P<0.05; Figure 5 5C2). These results suggest that DPPIV inhibition promotes EC cell progression from S and G2 phases to G1 phase.

### DPPIV knockdown reduces EC cell adhesion and induces apoptosis

To clarify the mechanism underlying DPPIV effects on cell growth, we examined apoptosis in cells by annexin V-PI staining. DPPIV knockdown in AN3CA cells reduced adhesion and increased the apoptosis rate to 19.5% (vs. 6.7% in the control group). This rate was reduced to 5.8% in cells overexpressing DPPIV (Figure 4B).

### DPPIV inhibition suppresses cell proliferation

Treatment with cisplatin for 72 h decreased AN3CA cell proliferation by 67% (Figure 4C), whereas DPPIV knockdown suppressed proliferation by 78% relative to controls (P<0.05). There were no synergistic effects associated with DPPIV knockdown and concurrent cisplatin treatment after 48, 72 or 96h (P>0.05; Figure 4). These results indicated that DPPIV is required for EC growth and DPPIV knockdown reduces cell proliferation.

### DPPIV overexpression increases tumorigenicity *in vivo*

To assess the tumor-forming capacity of DPPIV overexpressing cells *in vivo*, we injected transfected AN3CA cells (1×10^5^) into nude mice. DPPIV overexpression increased tumor-forming capacity as compared to untransfected and DPPIV-deficient cells. No tumors were observed in the DPPIV knockdown group eight weeks after injection (Figure 4D, Table 1).

**Table 1:** *In vivo* tumorigenicity of endometrial carcinoma cells (AN3CA)

Cell line	GROUP	Injection site	Tumor size (mm)			
			3W	5W	7W	8W
AN3CA	Control virus (for overexpress)	S.c.	0	1-2	4-5	8-9
	overexpression DPPIV	S.c.	3	10	Tumor has been removed	Tumor has been removed
	control virus(shRNA)	S.c.	0	0.5-2	3-4	7-9
	LV-shRNA	S.c.	0	0	0	0

### DPPIV activates HIF-1α-VEGFA signaling

**Figure 6:**
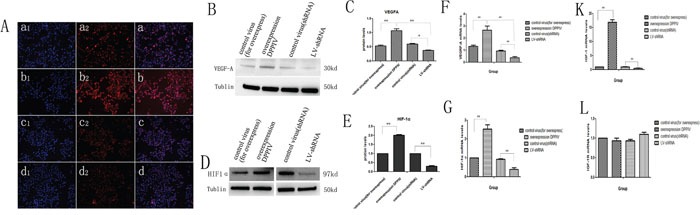
In AN3CA cells, DPPIV overexpression increase the protien and mRNA expression of HIF-1α and VEGFA; DPPIV overexpression inceases the mRNA expression of IGF-1, but not IGF-1R **A.** AN3CA cells after transfecte the vetor 96h, tained with anti VEGFA(1:400 ab51745, abcam) antibody, cell nucleus were stained with DAPI. Stained cells were viewed and imaged with immunofluorescence microscopy. **a** is the control virus-for overexpress; **b** is the overexpression DPPIV group; **c** is the control virus-for shRNA and d is the lentivirus-shRNA group (LV-shRNA). a1-d1 indicate nucleus stain; **a2-d2** indicate cytoplasm stain; **a-d** is the merge. **B, C.** VEGFA protien level was tested by western blot(WB) and quantify the protien expression levels of these four groups. **D, E.** HIF-1α protien level in the four groups was tested by western blot(WB) and analysis. **F-L.** VEGFA, HIF-1α, IGF-1 and IGF-1R mRNA level in the four groups was tested by Quantitative real-time RT-PCR analysis. Over expression DPPIV increase the VEGFA, HIF-1α, IGF-1 mRNA expression compare with the control group, but there is no obvious change in IGF-1R genes. Results are mean values ± SD (n = 4). (Student's t-test, (*p<0.05; **p<0.001). The graph is representative of three independent experiments.

We evaluated HIF-1α and VEGFA expression in transfected AN3CA cells. DPPIV overexpression in AN3CA cells increased HIF-1α and VEGFA protein and mRNA levels relative to controls. In contrast, HIF-1α and VEGFA expression was suppressed by DPPIV knockdown (P<0.05; Figure 6A–6G). These results suggested that DPPIV modulates EC cell proliferation and tumorigenesis via VEGFA signaling activation.

### DPPIV overexpression increases IGF-1, but not IGF-1R expression

Because DPPIV inhibitors have been used for treating diabetes and because insulin growth factor receptor (IGFR) signaling is important for EC oncogenesis, we speculated that DPPIV affects the IGFR pathway. We found that DPPIV overexpression increased IGF-1 mRNA levels by approximately 17-fold in AN3CA cells as compared to controls (P<0.05). However, there were no changes in IGF-1R expression (Figure 6K, 6L).

## DISCUSSION

Numerous cell types ubiquitously express DPPIV, including epithelial cells, fibroblasts and leukocytes. DPPIV has been implicated in the initial stages of malignant transformation and tumor progression, as well as immune regulation [14, 16, 26]. However, DPPIV also reportedly acts as a tumor suppressor [20–23] or oncogene [18, 19, 27, 28], and this may depend on the characteristics of individual cell lines and tumors. Understanding the mechanisms underlying DPPIV activity may clarify these contradictory findings [29, 30]. To this end, we examined the role of DPPIV in five EC cell lines and found that DPPIV modulated cell morphology, proliferation, invasiveness, apoptosis, tumorigenicity and via increased HIF-1α and VEGFA expression and signaling.

DPPIV is expressed at different levels in the five studied EC cell lines (AMEC, HEC1A, HEC50, Ishikawa, and RL95), which also exhibit varying degrees of differentiation. DPPIV overexpression in AMEC cells reduced cell proliferation rate, among other effects [25]. Other studies have linked DPPIV to cancer, diabetes [6–10] and obesity-related diseases [11–13]. We found that DPPIV expression was not correlated with ER1 or PR1 expression in EC cell lines. Similarly, in five human breast cancer specimens with high DPPIV expression included those that were positive or negative for ER [31]. In gastric cancer, where the four cell lines studied showed CD26 expression, while other two were negative [32, 33].

DPPIV overexpression increases blastocyst adhesion rates and trophectoderm outgrowth area [34]. We investigated whether DPPIV could impact oncogenesis-related cell functions via overexpression or knockdown in Ishikawa (ER- and PR-positive, well-differentiated), HEC-1B (ER- and PR-negative, moderately-differentiated) and AN3CA cells (ER and PR-negative, undifferentiated). Our results showed that DPPIV overexpression promoted cell proliferation and attachment to neighboring cells. Conversely, DPPIV knockdown induced apoptosis and loss of normal morphology in all cell lines, suggesting that DPPIV is a differentiation marker in human endometrial glandular cells [35]and plays a role in cell adhesion to the extracellular matrix [36–38].

DPPIV overexpression in EC cells not only facilitated tumor cell adhesion and promoted metastasis but also showed similar effect in Ishikawa, ANCA3 (low endogenous DPPIV expression) and HEC1-B (high endogenous DPPIV expression), indicating DPPIV's effect is irrelevant to ER, PR, differentiation state and endogenous expression. Elucidating the functions of DPPIV in cancer is complicated by the fact that most normal cell types exhibit DPPIV activity and that the enzyme has multiple functions. Neither the protease nor the cytoplasmic domain of the protein appears to be critical for its activity in tumor cells [20, 39]. In the issue of Nature Immunology, work by Barreira da Silva et al. [30], highlights the interaction between DPP4 and its substrate, chemokine CXCL10, to demonstrate the function of DPP4-mediated post-translational modification of chemokines in regulating tumor immunity. Our study also indicated that DPPIV is not only a differentiation marker but also plays an very important role in maintaining cell's cytoskeleton, regardless of its expression level. In the future study, we will continue to provide more clear evidence of role for DPP4 in tumor biology and its likely interaction with the tumor microenvironment.

Tumor growth was delayed in DPPIV-null as compared to control mice, and DPPIV inhibition by sitagliptin treatment reduced tumor growth [7]. To date, five gliptins that vary in their pharmacodynamic and pharmacokinetic properties have been approved for clinical use [1, 7, 10]. In our study, 1 mM sitagliptin decreased AN3CA cell proliferation by 60% and blocked invasion and cell cycle progression. Additional *in vivo* studies are needed to confirm the effects of sitagliptin in EC.

EC is often confined to the endometrium without myometrial invasion or lymph node metastasis, and can be treated by hysterectomy and bilateral salpingo-oophorectomy with a 5-year survival rate of 96%. However, survival is poor in recurrent or metastatic EC, with a 5-year survival rate of only 17% [4], and these patients receive adjunctive platinum-based chemotherapy (e.g., cisplatin and doxorubicin or carboplatin and paclitaxel). The relationship between DPPIV and chemotherapy resistance in CSCs has been investigated in colorectal cancer, in which higher DPPIV levels were observed in cells with induced resistance to cisplatin. These cells also showed upregulation of the differentiation markers, CD133 and CD44, along with DPPIV [19, 38], suggesting that high DPPIV levels are associated with resistance to chemotherapy. EC includes CSCs that are capable of self-renewal and differentiation [40]. Endometrial CSCs are enriched in EC [41] and are related to CSCs in other tumor types [40]. Colorectal cancer CSCs, co-express CD133 and DPPIV, which may indicate de-differentiation and metastasis [19]. Consisted with others [43], we did not find any correlation between DPPIV and CD133 expression (data not shown) in CD133-positive EC cells [42]. Additional studies are needed to identify any possible correlations between DPPIV and CD44 in EC. In testing resistance to chemotherapy, our study showed that cisplatin treatment, with or without DPPIV knockdown, inhibited tumorigenesis, suggesting that DPPIV depletion could reduce tumor burden in EC. We observed no synergistic effects associated with DPPIV knockdown and concurrent cisplatin treatment.

VEGF-targeted therapy alone or combined with chemotherapy is used to treat many cancers [44]. VEGFA-mediated angiogenesis during the epithelial-to-mesenchymal transition has been proposed as a link between cancer stemness and tumor initiation [45]. DPPIV is expressed in microvascular endothelial cells of various human tissues such as liver, spleen, lung, brain and heart, as well as in human vascular smooth muscle cells [46, 47]. The HIF-1α-VEGFA signaling pathway is the best studied with respect to hypoxia/ischemiainduced angiogenesis regulation [48]. HIF-1α is dynamically regulated. In normoxic conditions, HIF-1α can be hydroxylated and then quickly degraded by the von Hippel-Lindau E3 ubiquitin ligase complex. In the absence of oxygen, HIF-1αhydroxylation is blocked. HIF-1α protein accumulates and translocates into the nucleus to form a transcriptional complex with HIF-1β, p300and CREB (cAMP response element)-binding protein, initiating the transcription of many genes, including the VEGF family.

HIF-1α is increasingly recognized as playing broad and critical roles in normal development, postnatal physiology, cancer and many other diseases [49–51]. HappMolitoris, *et. al* found that inhibition of HIF-1α degradation unmasks estradiol induction of VEGF expression in ECC-1 cancer cells *in vitro* [52]. Given the critical role of HIF-1α in proangiogenic signaling, we assessed HIF-1αand VEGFA expression andsignaling after DPPIV overexpression or knockdown. DPPIV overexpression increased, and knockdown decreased HIF-1α levels in AN3CA cells. DPPIV can therefore alter VEGFA expression and activate the VEGFA signaling through HIF-1α. This is the first report of DPPIV modulating EC progression via HIF-1α-VEGFA signaling.

The potential link between the insulin/IGF-I signaling pathways and cancer has been the focus of much investigation over the last several years [53, 54]. IGF-I activityismediated by the IGF-I receptor (IGF-IR). IGF-IR gene expression regulation is mainly mediated at the level of transcription. Several studies have shown a correlation between IGF components and endometrial cancer risk [55, 56]. We found that IGF-1 expression increased in AN3CA cells overexpressing DPPIV, while IGF-1R expression was unchanged.

This study was the first to demonstrate that DPPIV overexpression in EC cells altered cell morphology, promoted cell proliferation, invasion and tumorigenesis, and inhibited apoptosis, and that DPPIV inhibition resulted in the opposite effects. DPPIV acted through regulation of HIF-1α-VEGFA signaling. DPPIV overexpression increased IGF-1, but not IGF-1R expression. Taken together, our results suggest that DPPIV is a promising therapeutic target for EC treatment.

## MATERIALS AND METHODS

### Cell lines and reagents

The EC cell line, AN3CA (an undifferentiated malignant cell line), was generously donated by Dr. Weiwei Feng (Hospital and Institute of Obstetrics and Gynecology, Fudan University Shanghai Medical College, Shanghai, China) and Dr. GB Mills (M. D. Anderson Cancer Center, Houston, TX, USA), HEC-1-B and KLE cells (moderately and poorly-differentiated cell lines, respectively) were purchased from the American Type Culture Collection (ATCC® HTB113™ and ATCC® CRL1622L, respectively; Manassas, VA, USA). Ishikawa cells (well-differentiated cell line) and HEC-1-A (moderately-differentiated cell line) were purchased from Fudan IBS Cell Center (FDCC-HZC067 and FDCC-HZC069, respectively; FDCC, Shanghai, China). Cells were obtained in 2015 and passaged in our laboratory for fewer than six months after receipt or resuscitation. Cells were maintained in Roswell Park Memorial Institute 1640 (RPMI) medium (Gibco, Grand Island, NY, USA), Eagle's minimal essential medium (EMEM) (ATCC), or Dulbecco's Modified Eagle's Medium (DMEM)/F12 (Gibco) containing 10% fetal bovine serum (Gibco) in a humidified atmosphere with 5% CO_2_ at 37°C. Culture plates and dishes were purchased from Corning (Corning, NY, USA). Unless otherwise specified, all other reagents were from Sigma (St. Louis, MO, USA).

### Cell morphology was observed by inverted microscope, ER1 and PR1 expression was determinated via qRT-PCR

We confirmed ER1 and PR1 expression in all EC cell lines via qRT-PCR as compared with analogous data provided by ATCC. EC cells morphologies before and after DPPIV up- or downregulation were also recorded using an IX71 microscopy system coupled to a DP73 digital camera (both from Olympus, Tokyo, Japan).

### Flow cytometry

DPPIV expression in cells was determined using a FACSVerse flow cytometer (BD Biosciences, San Jose, CA, USA). Cells were cultured in 6-well plates, trypsinized and centrifuged to obtain a pellet, and phycoerythrin (PE)-conjugated anti-DPPIV antibody (12-0269; eBioscience, San Diego, CA, USA) was added to each tube. After a 20-min incubation at 4°C under protection from light, cells were centrifuged and resuspended in phosphate-buffered saline (PBS). IgG served as a negative control.

### Lentiviral shRNA vector construction and transfection

Three short hairpin (sh)RNAs against human DPPIV were designed with the following sequences: shRNA#1: 5'-CCA ATT TAA CGA CAC AGA A-3', shRNA#2: 5'-CTG AAG TTA TAC TCC TTA A-3', and shRNA#3: 5'-CAC TTA TTG AAT ACT CCT T-3'. Oligonucleotides encoding shRNA sequences and one negative control sequence (5'-TTC TCC GAA CGT GTC ACG T-3') were synthesized and annealed. Double-stranded inserts were subcloned into HpaI/XhoI restriction sites of the lentiviral vector, pFU-GW-RNAi, encoding green fluorescent protein (GFP) (Genechem, Shanghai, China), which was transformed into *Escherichia coli* cells. Positive recombinant clones were selected via PCR. Recombinant non-integrative lentiviral vectors were produced by co-transfecting 293T cells with the lentivirus (LV) expression and packaging plasmids using Lipofectamine 2000 (Invitrogen, Carlsbad, CA, USA). Ishikawa, HEC-1B and AN3CA cells were infected at various multiplicities of infection (MOI = 1, 10, 20 and 50); 72–96 h later, transduction efficiency was verified under a fluorescence microscope and by western blotting and quantitative real-time (qRT-) PCR (Supplementary Figure 3&4). The MOI=20 and shRNA#2 were identified and used for experiments. Cells were divided into two groups: control shRNA (transfected with negative control virus) and a LV-shRNA (transfected with target shRNA lentivirus). Cells were used in experiments within two passages of establishing DPPIV-knockdown.

### DPPIV overexpression vector construction and transfection

A lentiviral vector expressing the DPPIV coding sequence was constructed by GeneChem (Shanghai, China) and used for DPPIV overexpression. Ishikawa, HEC-1B and AN3CA cells were infected at various MOI (1, 10, 20 and 50); 72–96 h later, DPPIV expression was visualized under a fluorescence microscope and detected by western blotting and qRT-PCR (Supplementary Figure 3&4). Cells were divided into two groups: control (transfected with control virus) and DPPIV overexpression (transfected with DPPIV lentiviral vector). Cells were used in experiments within two passages of establishing DPPIV overexpression.

### Cell proliferation assay

Ishikawa, HEC-1B and AN3CA cells were transfected with DPPIV overexpression or shRNA vectors; 96 h later, cells were trypsinized and centrifuged to obtain a pellet, and were seeded in 96-well plates (4×10^3^ cells/well) for 72 or 96 h. The AN3CA cell line was used for the DPPIV inhibitor experiment. Cells were seeded in 96-well plates (4×10^3^ cells/well) and treated 24 h later with different concentrations of the DPPIV inhibitor, sitagliptin phosphate (1, 2, 4 or 8 mM) (#13252, Cayman Chemical, Ann Arbor, MI, USA), for 72 h at 37°C. Cell viability was assessed using Cell Counting Kit-8 (Dojindo Laboratories, Kumamoto, Japan) 24 h after inhibitor removal. Absorbance was measured at 450 nm using a microplate reader.

### Cell migration assay

Uniform wounds were introduced into cell cultures using a culture insert (#80201; ibidi, Munich, Germany). The inserts were placed in individual wells of a six-well plate. Ishikawa, HEC-1B and AN3CA cells transfected with vector for 96 h or AN3CA cells treated with sitagliptin or left untreated were seeded in each reservoir of the insert (1×10^4^cells in a finalvolume of 100 μl). Inserts were removed after cells adhered. The gaps (wounds) were washed with serum-free medium, and 2ml fresh medium were added to each well. AN3CA cells were treated with 1 mMsitagliptin, with culture medium aloneserving as a control. Cell migration into the wound area was recorded using an IX71 microscopy system coupled to a DP73 digital camera (both from Olympus, Tokyo, Japan) at 0 and 48 h. Wound closure at 24–48 h was compared with time 0.

### Cell cycle analysis

The four groups of AN3CA cells 96 h after transfection, the untransfected cells treated with sitagliptin, and the cells left untreated were seeded in 6-well plates at 2×10^5^ cells/well for 48 h. Cells were collected and fixed in 75% methanol, and then stored at -4°C overnight. 24 h later, cells were washed with PBS, centrifuged and resuspended in 50 μl RNase A solution (250 μg/ml) containing 10 mM Tween 20 and 50 μlpropidium iodide (PI), followed by incubation in the dark for 30 min at 37°C. Labeled cells were analyzed by flow cytometry on a FACSCalibur instrument (Becton Dickinson, Franklin Lakes, NJ, USA).

### Annexin V-PI apoptosis assays

Apoptotic cells were quantified by surface annexin V-PI staining. AN3CA cells (2×10^5^ cells/group) after 96 h transfection and untransfected cells with or without 48 h sitagliptin treatment were collected using 0.05% trypsin, with the supernatant used to terminate the digestion. Cells were washed with PBS and resuspended in binding buffer composed of 10 mmol/l HEPES (pH 7.4), 2.5 mmol/l CaCl_2_ and 140 mmol/l NaCl. 195 μl Apoptosis Assays Buffer was added to cells, followed by incubation with 5 μl annexin V-PI (C1065; Beyotime Institute of Biotechnology, Shanghai, China) for 15 min in the dark at room temperature. A total of 10,000 cells were acquired per sample and data were analyzed using Cell Quest software (BD Pharmingen, San Jose, CA, USA).

### Chemotherapy sensitivity assay

The effect of cisplatin chemotherapy was evaluated in AN3CA cells transfected with vector or left untransfected for 96 h. Cells were seeded at 4000/per well in a 96-well plate in 100 μl DMEM/F12; 24 h later, cells were treated for 96 h with 10 μmol cisplatin (P4394; Sigma). Viability was assessed using Cell Counting Kit-8. Absorbance was measured at 450 nm using a microplate reader.

### Animal studies

Six-week-old BALB/c nude mice were randomly divided into four groups (n=6 each): control virus, DPPIV overexpression, control shRNA virus and LV-shRNA. Animals were maintained under standard conditions according to institutional guidelines. Each mouse was injected subcutaneously in the flank with 1×10^5^ AN3CA cells resuspended in serum free-DMEM/Matrigel (BD Pharmingen) at a 1:1 ratio. Tumor volume (mm^3^) was monitored weekly after inoculation and was estimated using the following formula: width × length. Tumorigenicity experiments were terminated eight weeks after cell injection. In mice without visible tumor nodules, an incision was made at the injection site to determine whether a tumor had formed. Animal experiments were approved by the Institutional Animal Care and Use Committee.

### Immunocytochemistry

AN3CA cells (1×10^4^/group) transfected with vector for 96 h were fixed and stained with anti-VEGFA antibody (1:400, ab51745; Abcam, Cambridge, MA, USA). After three washes with PBS, cells were incubated with PE-conjugated goat anti-rabbit IgG (Santa Cruz Biotechnology, Santa Cruz, CA, USA). Nuclei were visualized viaDAPI staining and cells were observed under a fluorescence microscope.

### Western blot analysis

Total protein was extracted from EC cells using buffer containing Halt Protease Inhibitor Cocktail (#87786, #78429, and #78438; Thermo Fisher Scientific, Waltham, MA, USA). Equal amounts of protein (30 μg) were separated by 10–12% sodium dodecyl sulfate polyacrylamide gel electrophoresis (SDS-PAGE) and transferred to a polyvinylidene fluoride (PVDF) membrane, which was blocked with 5% non-fat milk and incubated overnight at 4°C with antibodies against DPPIV (1:400, ab28340), HIF-1α (1:500, ab463), VEGFA (1:400, ab51745) (Abcam) or β-tubulin (1:1000, #2128; Cell Signaling Technology, Danvers, MA, USA). Membranes were then incubated with a peroxidase-conjugated secondary antibody for 2 h at room temperature. Immunoreactivity was visualized by enhanced chemiluminescence using an Alpha Innotech Imaging System (Protein Simple, Santa Clara, CA, USA). Protein band intensity was normalized to the level of β-tubulin. Each experiment was repeated at least three times.

### qRT-PCR

Total RNA was extracted from cell lines with TRIzol reagent (Invitrogen) and DPPIV, HIF-1α, VEGFA, IGF-1 and IGF-1R mRNA levels were quantified via qRT-PCR (271001463; Applied Biosystems, Foster City, CA, USA) using FastStart Universal SYBR Green Master Mix (#13396700; Roche, Indianapolis, IN, USA). Primer sequences are specified in Supplementary Table 1. Expression was normalized for the endogenous reference GAPDH gene.

### Statistical analysis

All *in vitro* studies were carried out in triplicate and results are expressed as means ± SD. Statistical significance between means was evaluated with the Student's t test or by analysis of variance (ANOVA) for multiple comparisons. Significance was defined as P<0.05.

## SUPPLEMENTARY MATERIALS FIGURES AND TABLES


