# Supplementary material for: DPPIV promotes endometrial carcinoma cell proliferation, invasion and tumorigenesis
Source: Oncotarget. 2017 Jan 2;8(5):8679–92. doi: 10.18632/oncotarget.14412 (PMC5352432; doi:10.18632/oncotarget.14412)
Supplement: Supplementary file 1 [file oncotarget-08-8679-s001.pdf]

DPPIV promotes endometrial carcinoma cell proliferation, invasion and tumorigenesis

SUPPLEMENTARY FIGURES AND TABLE

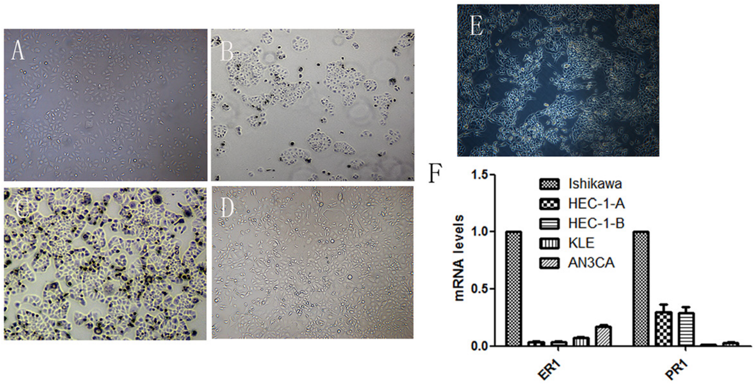

Supplementary Figure 1: EC cell morphology was observed by inverted microscope and quantitative analysis of the percentages of cells expressing ER1 and PR1 mRNA. A–E. EC cell morphology was recorded using an IX71 microscopy system coupled to a DP73 digital camera. (**A,** Ishikawa; **B,** HEC1-A; **C,** HEC1-B; **D,** KLE; and **E,** AN3CA). HEC1-A, HEC1-B, and KLE cells showed an epithelioid pattern in contrast to cells expressing low levels of DPPIV (Ishikawa and AN3CA), which show a spindle/bipolar fibroblast-like pattern. **F.** Quantitative analysis of ER1 and PR1 mRNA expression as determined by qRT-PCR. Ishikawa cells were PR1- and ER1-positive and other cell lines were negative for expression of the two receptors.

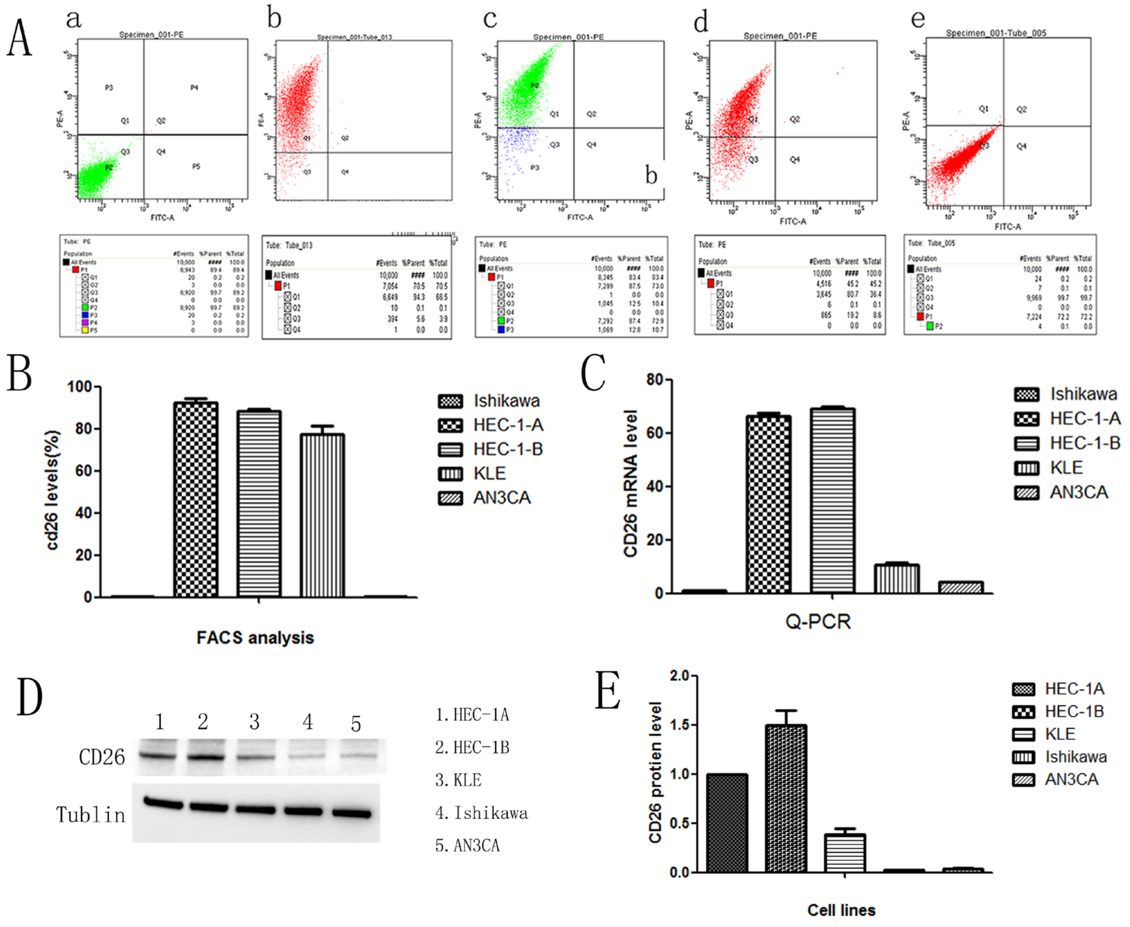

Supplementary Figure 2: DPPIV expression in EC cell lines by flow cytometric analysis, qRT-PCR and Western Blotting(WB). A. Flow cytometric analysis of DPPIV expression in EC cell lines (a, Ishikawa; b, HEC1-A; c, HEC1-B; d, KLE; and e, AN3CA). **B.** Fraction of DPPIV-positive cells quantified based on five individual cell sorting experiments with each cell line. **C.** qRT-PCR analysis of DPPIV expression in EC cell lines. **D.** DPPIV protien levels in five cell lines was determined by WB. **E.** Quantitative analysis of the WB results shown in E.

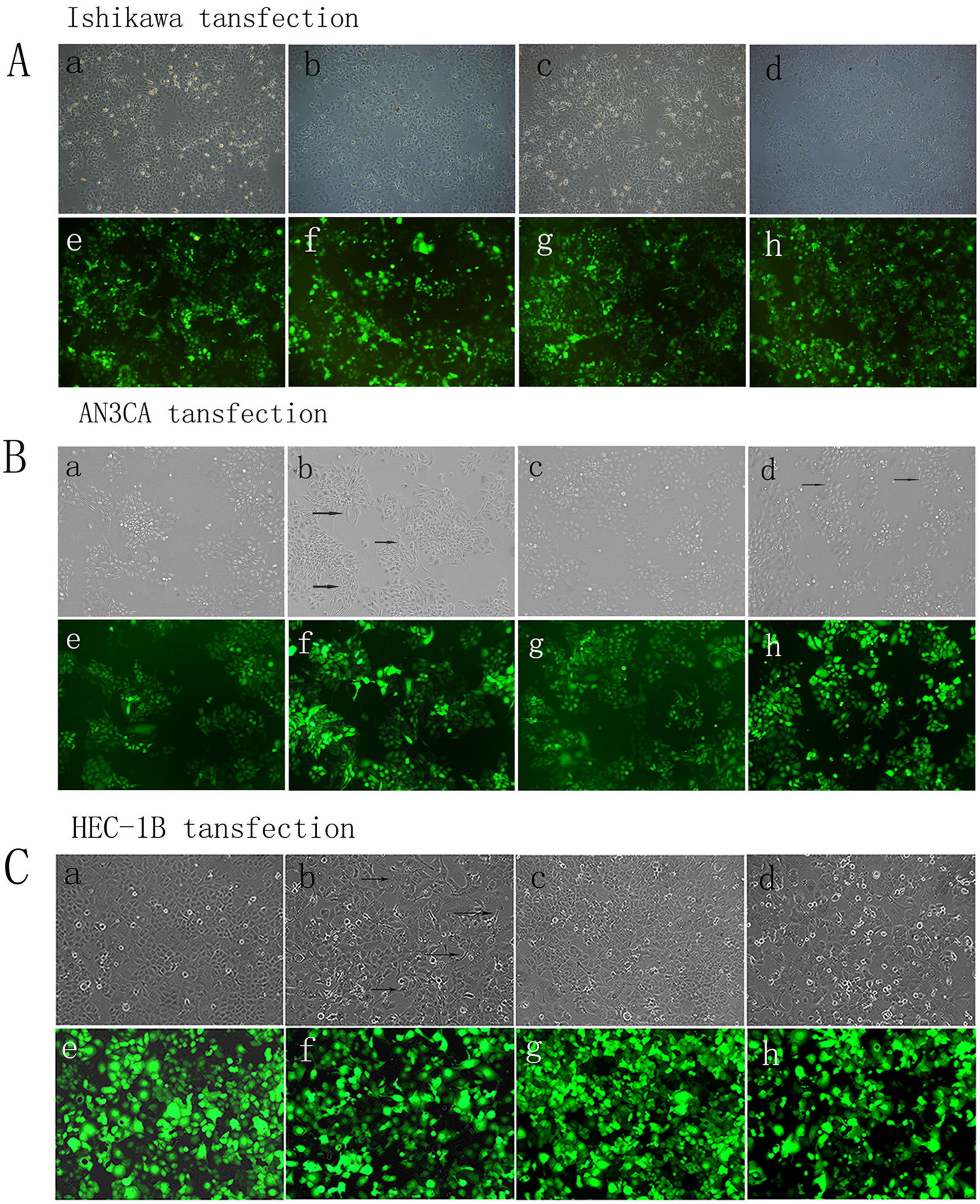

Supplementary Figure 3: LV and shRNA transduction efficiency in Ishikawa, HEC-1B and AN3CA cells by a fluorescence microscope. A. Ishikawa cells; **B.** AN3CA cells; **C.** HEC-1B cells a and e, overexpression control; b and f, DPPIV overexpression; c and g, shRNA control; d and h, LV-shRNA.

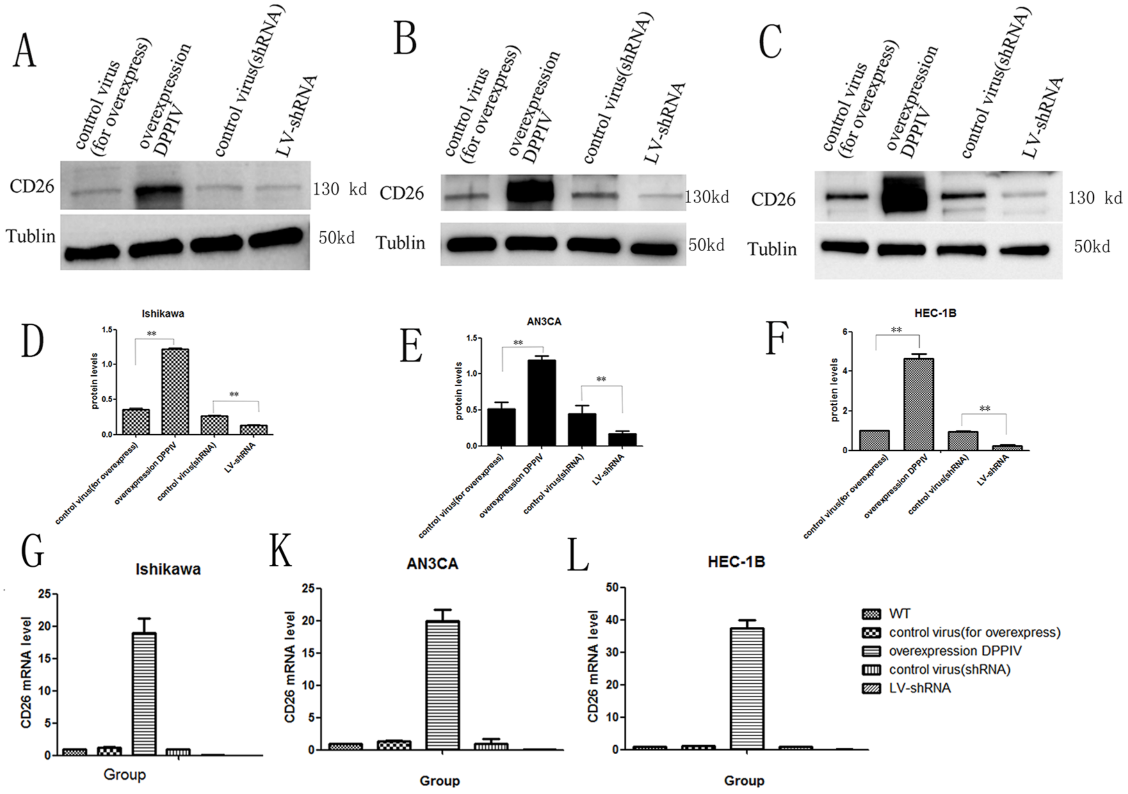

Supplementary Figure 4: LV and shRNA transduction efficiency in Ishikawa, HEC-1B and AN3CA cells by Western Blotting(WB) and qRT-PCR. A-C. The transduction efficiency in Ishikawa, HEC-1B and AN3CA cells, was determined by WB. **D-F.** Quantitative analysis of the WB results shown in A to C. **G-L.** qRT-PCR analysis of DPPIV expression in Ishikawa, HEC-1B and AN3CA cells. Bars represent mean ± SD of three experiments. $*P < 0.05$; $**P < 0.001$, analysis of variance followed by Tukey's test).

Supplementary Table 1: Summary of PCR primers

Gene name	Primer sequences
DPPIV	5'-CGG TCC TGG TCT GCC CCT CTA-3';<br>3'-CGC CAC GGC TAT TCC ACA CTT-5'
HIF-1α	5'-GTCGGACAGCCTCACCAAACAGAGC-3';<br>3'- GTTAACTTGATCCAAAGCTCTGAG-5'
VEGFA	5'-CTC AGA GCG GAG AAA GCA TTTG-3';<br>3'-TTA ACT CAA GCT GCC TCG CCT-5'
IGF-1	5'-CAT GTC CTC GCA TCT CT-3';<br>3'-ATA CCC TGT GGG CTT GTT GA-5'
IGF-1R	5'-GGG AAT GGA GTG CTG CTG TAT G-3';<br>3'-CAC AGA AGC TTC GTT GAG AA-5'
GAPDH	5'-GGA CCT GAC CTG CCG TCT AG-3';<br>3'-GTA GCC CAG GAT GCC CTT GA-5'
